# Cytokinesis in *Trypanosoma brucei* differs between bloodstream and tsetse trypomastigote forms: implications for microtubule‐based morphogenesis and mutant analysis

**DOI:** 10.1111/mmi.12436

**Published:** 2013-11-15

**Authors:** Richard J. Wheeler, Nicole Scheumann, Bill Wickstead, Keith Gull, Sue Vaughan

**Affiliations:** ^1^ Department of Biological and Medical SciencesFaculty of Health and Life ScienceOxford Brookes UniversityOxfordOX3 0BPUK; ^2^ Sir William Dunn School of PathologyUniversity of OxfordSouth Parks RoadOxfordOX1 3REUK; ^3^ School of Life SciencesUniversity of NottinghamMedical SchoolQueen's Medical CentreNottinghamNG7 2UHUK

## Abstract

Trypanosomes use a microtubule‐focused mechanism for cell morphogenesis and cytokinesis. We used scanning electron and video microscopy of living cells to provide the first detailed description of cell morphogenesis and cytokinesis in the early‐branching eukaryote *Trypanosoma brucei*. We outline four distinct stages of cytokinesis and show that an asymmetric division fold bisects the two daughter cells, with a cytoplasmic bridge‐like structure connecting the two daughters immediately prior to abscission. Using detection of tyrosinated α‐tubulin as a marker for new or growing microtubules and expression of XMAP215, a plus end binding protein, as a marker for microtubule plus ends we demonstrate spatial asymmetry in the underlying microtubule cytoskeleton throughout the cell division cycle. This leads to inheritance of different microtubule cytoskeletal patterns and demonstrates the major role of microtubules in achieving cytokinesis. RNA interference techniques have led to a large set of mutants, often with variations in phenotype between procyclic and bloodstream life cycle forms. Here, we show morphogenetic differences between these two life cycle forms of this parasite during new flagellum growth and cytokinesis. These discoveries are important tools to explain differences between bloodstream and procyclic form RNAi phenotypes involving organelle mis‐positioning during cell division and cytokinesis defects.

## Introduction

Protists are usually immediately recognizable by their cell shape. The replication of this shape through a cell cycle involves a series of cellular morphogenetic events and then a cytokinesis event. Two types of cell cycle are often recognized – the proliferative type, which leads to two daughter cells with the same characteristics as the original, and the differentiation type, which leads to the formation of at least one daughter cell which differs from its parent. The former type of proliferative cell cycle was historically referred to as ‘binary fission’. However, it is now clear that the cellular morphogenetic process leading to the formation of two seemingly identical daughters often involves asymmetry in the formation and positioning of structures within the dividing cells and resulting daughters. Examples of this can be found in the cell division cycles of *Saccharomyces cerevisiae* and *Schizosaccharomyces pombe* (for reviews see Pollard and Wu, 2010; Oh and Bi, 2011). For most other protists although there are excellent descriptions of cell shape, form and cytoplasmic organization little is known on how these are reproduced throughout the cell cycle and the nature and inheritance patterns of the two daughter cells. In this study we provide detailed analysis of such events in two important proliferative life cycle stages of the African trypanosome *Trypanosoma brucei*, a notable pathogen and an example of a protist which achieves cytokinesis with a cytoskeleton almost entirely composed of microtubules.

Underlying the *T. brucei* plasma membrane is the sub‐pellicular microtubule array, which defines the characteristic shape of trypanosomes (Vickerman, 1962; Anderson and Ellis, 1965; Sherwin and Gull, 1989b). This microtubule cytoskeleton remains intact during all stages of the cell cycle; thus the two daughter cells are produced within the confines of this array and the array must undergo significant spatial and temporal re‐modelling (Sherwin and Gull, 1989b). Animal cells use the actinomyosin contractile ring to drive in‐folding of the plasma membrane (called furrow ingression) to delineate two daughters, and genomic analysis indicates it is likely the genes for this process were present in the common ancestor of all eukaryotes. However, the bikonts (which include trypanosomatids) lack myosin‐II (Richards and Cavalier‐Smith, 2005; Baluska *et al*., 2006; Foth *et al*., 2006) and RNAi knockdown of actin in *T. brucei* procyclic (tsetse) form trypomastigotes indicates that it is not essential for division (García‐Salcedo *et al*., 2004). The mechanism by which cytokinesis is achieved must, therefore, differ significantly from the well‐analysed metazoa (Farr and Gull, 2012). Actinomyosin‐independent cytokinesis is observed in plants which also lacks myosin II, where there is no in‐folding of membrane; instead membrane fusion events at the microtubule‐rich phragmoplast achieve formation of two separate plasma membranes (Müller *et al*., 2009). Addressing how cytokinesis is achieved in a bikont protist and whether it is similar to actinomyosin‐independent cytokinesis of plants, is therefore of general biochemical interest in understanding how cytokinesis is achieved in more diverse organisms.

*Trypanosoma brucei* undergoes a complex life cycle; alternating between proliferating and non‐proliferating life cycle forms during transit between the tsetse fly vector and mammalian host (for review see Steverding, 2008). Proliferative and differentiation division events must occur throughout its life cycle to guarantee survival, adaptation and transmission. Following the discovery of *T. brucei* as the causative agent of sleeping sickness and nagana in sub‐Saharan Africa, techniques for axenic culture of two of the proliferating life cycle stages, the procyclic form (found in the midgut of the tsetse fly) and the mammalian bloodstream form, were developed and are widely used in cell and molecular studies of *T. brucei* biology, life cycle stage differentiation, pathogenicity, and as a model organism for analysis of the flagellum and cytoskeleton. In our previous work, we established descriptions of cell cycle, cytokinesis and cellular morphogenesis in the procyclic form of *T. brucei* (Sherwin and Gull, 1989a; 1989b; Robinson *et al*., 1995). We demonstrated that the sub‐pellicular array is not disassembled during division and that new microtubules are added to the existing cortex as the cell cycle progresses, with both daughter cells inheriting a mixture of old and newly assembled microtubules (Sherwin *et al*., 1987; Sherwin and Gull, 1989b; Woodward and Gull, 1990). Using pharmacological insults, we revealed that trypanosomes possessed unusual ‘dependency’ relationships between cellular structures and events during the cell cycle – a fact that has been confirmed and extended by others (Ploubidou *et al*., 1999; Tu and Wang, 2004; Hammarton *et al*., 2007). Morphogenesis in the cell cycle therefore involves both semi‐conservative (sub‐pellicular array) and conservative (flagellum, flagellum attachment zone, basal body) inheritance patterns (for review see Gull, 1999). However, how this division process compares with the bloodstream form life cycle stage, where there are many known organelle and biochemical differences (Vickerman, 1962; 1969; Anderson and Ellis, 1965), and precisely how the microtubule cytoskeleton achieves cytokinesis in either life cycle stage are not known.

The application of RNAi technologies in *T. brucei* has led to a rapidly increasing number of mutant phenotypes being described, often including failure in cell division. Given the growing importance of these post genomic studies we were struck by the fact that there has been no concerted attempt at a careful analysis of cytokinesis in trypanosomes or a discrete comparison of the morphogenesis process and cytokinesis between the two most studied proliferative forms – bloodstream and procyclic form trypomastigotes. Analyses of RNAi knockdowns of the same protein often reveal phenotype differences between the two major life cycle forms (e.g. Hammarton *et al*., 2003; Tu and Wang, 2004; Kumar and Wang, 2005; Broadhead *et al*., 2006; Rothberg *et al*., 2006), suggesting some differences. Studies vary tremendously in the depth of analysis but nevertheless there is a general theme of variation between the two major trypomastigote forms and in the types of cytokinetic mutant phenotypes produced. The lack of a comparative analysis of overall cellular morphogenesis during the cell cycle and the cytokinesis process is a serious hindrance to meaningful interpretation of such mutant phenotypes.

Here we provide a comparative analysis of division and cytokinesis analysis using fluorescence markers, electron and live cell microscopy outlining the major morphogenetic stages of the cell cycle in both procyclic and bloodstream forms to identify the shared and distinct mechanisms used by both life cycle stages to achieve cytokinesis. We show that an asymmetric division fold bisects the two daughter cells, the placement of which differs in the two life cycle stages, and a discrete cytoplasmic bridge connects the two daughter cells immediately prior to abscission. We have dissected the underlying spatial and temporal re‐modelling of the microtubule cytoskeleton using markers for new or growing microtubules and location of a plus end binding protein XMAP215. These demonstrate spatial asymmetry in the underlying microtubule cytoskeleton throughout the cell division cycle and identify the major regions of growth and reorganization. These discoveries and differences between the two life cycle forms have implications for both understanding cytokinesis in an organism with a microtubule dominated cytoskeleton and past and future studies on the differing phenotypes in cell morphogenesis in the procyclic and bloodstream form stages of this parasite.

## Results

### Procyclic and bloodstream forms have related but distinct morphogenetic events leading to cytokinesis

To compare the morphological changes during division of these two life cycle stages we performed a comprehensive analysis by SEM. In order to identify and characterize minor stages in the cell cycle many thousands of images were examined for each cell form and categorized by at least two independent observers.

### Cell division in the procyclic form

**Figure 1:**
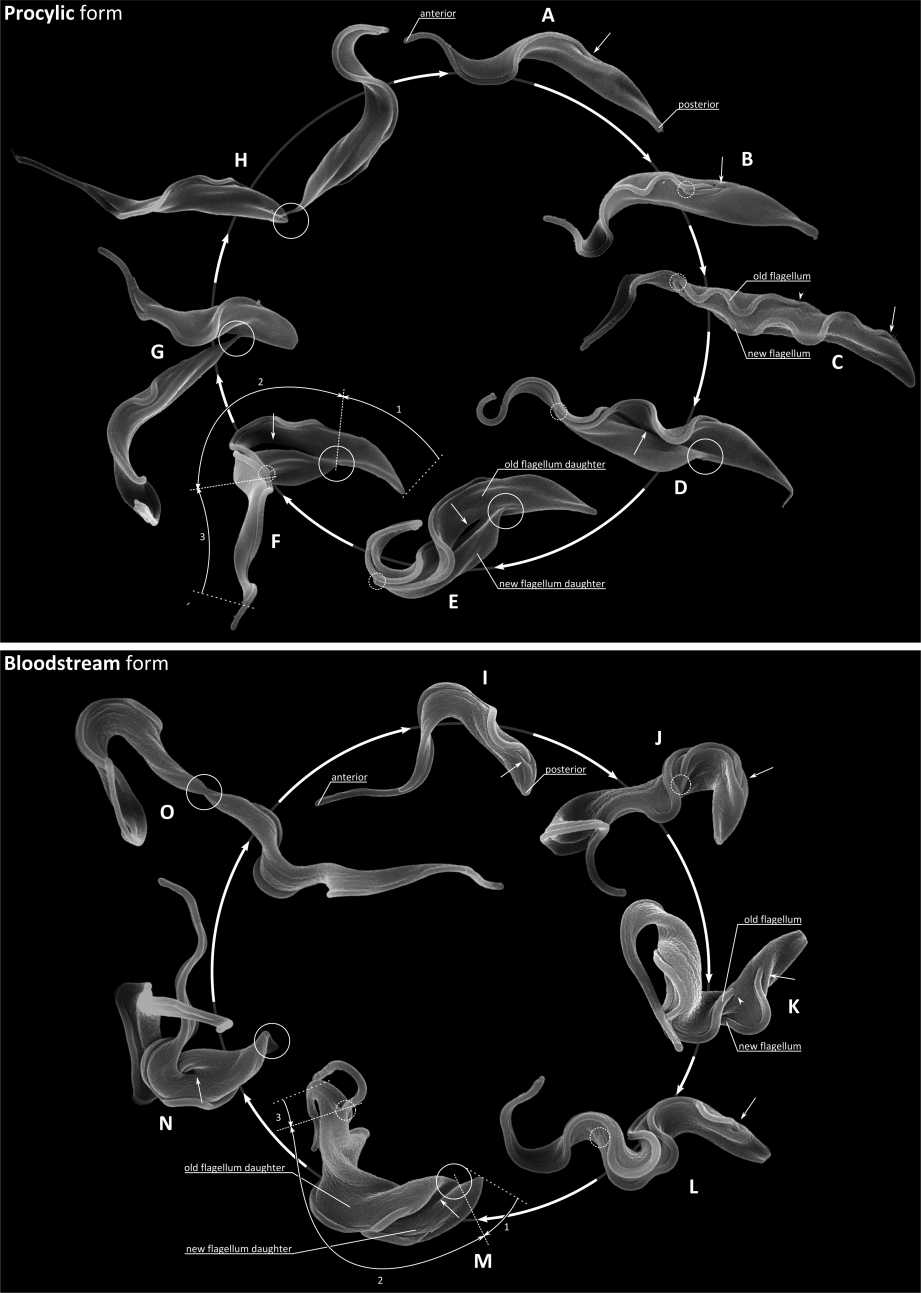
Differences in external morphology during the cell division cycle of the procyclic and bloodstream forms*.* Composite scanning electron micrograph images of stages of procyclic (A–H) and bloodstream (I–O) form cell division. A and I. G1 cell with a single attached flagellum. The exit point of the flagellum from the flagellar pocket is arrowed and the anterior and posterior indicated. B and J. A new flagellum has grown to extend from the flagellar pocket (arrow). The distal tip of the new flagellum is laterally connected to the old flagellum in the procyclic (B) and is laterally embedded in the groove structure in the flank of the cell in the bloodstream form (J), indicated by dashed circles. C, K–L. The flagellar pocket associated with the new flagellum (arrowed) is positioned posterior to the flagellar pocket associated with the old flagellum (arrowhead). The new flagellum is located to the left of the old when viewed looking from posterior to anterior. D and M. A division fold is evident between the two flagella (arrowed) which is located along the long axis and begins to define the daughter cell shape. There are two distinct posterior end profiles. The new‐flagellum daughter inherits the existing posterior end and a new posterior end is formed for the old‐flagellum daughter (circled). The new flagellum is still attached to the old by the flagella connector in the procyclic form (D), but has grown free of the cell body in the bloodstream form (M), indicated by dashed circles. E–F, N. A division cleft has opened up between the daughters (arrow) and the new flagellum tip remains attached to the old flagellum by the flagella connector in the procyclic form (E–F). G–H, O. Pre‐abscission stage. In the procyclic form (G–H) the two daughter cells are attached by the posterior end of the old‐flagellum daughter (circled) to the side of the new‐flagellum daughter by a cytoplasmic bridge connection. In the bloodstream form (O) a posterior‐to‐posterior (circled) configuration is typical.

Procyclic trypanosomes have a long slender form with a single flagellum that exits the flagellar pocket at the posterior end of the cell and is attached along the length of the cell body (Fig. 1A). The first external indication of cell cycle progression is a new flagellum that exits the flagellar pocket and whose tip is connected to the old flagellum (arrow) via a transmembrane mobile junction – the flagella connector (FC) (Fig. 1B) (Moreira‐Leite *et al*., 2001; Briggs *et al*., 2004; Davidge *et al*., 2006). This connection is maintained as the new flagellum grows in an anterior direction and the two flagellar pockets segregate (Fig. 1C). The exit point from the flagellar pocket of the new flagellum (Fig. 1C; arrow) is always positioned posterior to that of the old flagellum (arrowhead) along the long axis of the cell. The new flagellum is always positioned to the left of the old when viewed from cell's posterior end (Sherwin and Gull, 1989b) (Fig. 1C). During this time the flagella begin to segregate, and are widely separated around the circumference of the cell near the posterior but more closely adjacent towards the anterior (Fig. 1C). Here we define four characteristic stages of cytokinesis: division fold generation, division furrow ingression, pre‐abscission and abscission. In procyclics the division fold is an invagination of the cell body between the two flagella, which facilitates the early definition of two nascent daughter cells (Fig. 1D; arrow). The fold does not extend all the way to the posterior end of the dividing cell, but stops some way back from this (Fig. 1D and E; circled). This defines two nascent posterior ends; a nascent posterior end associated the daughter cell with the new flagellum (called the new‐flagellum daughter) (Fig. 1E) and a nascent posterior end associated with the daughter cell with the old flagellum (called the old‐flagellum daughter) (Fig. 1E). Even though the fold is placed asymmetrically along only a portion of the original cell, it does bisect the cell approximately by volume (Fig. 1F and G) yielding daughter cells of similar but not identical shape (see later). The second stage in cytokinesis is division furrow ingression characterized by the appearance of a gap between the two daughters (Fig. 1E; arrow). The distal tip of the new flagellum remains attached to the old via the flagella connector during this process (Briggs *et al*., 2004) (Fig. 1E and F; dashed circle) and the new flagellum has not grown to reach the anterior of the existing cell body (Davidge *et al*., 2006). In the third stage, pre‐abscission, the two nascent daughter cells remain connected via a thin section of membrane linking the posterior end of the old‐flagellum daughter cell (Fig. 1G; circled) with the side of the new‐flagellum daughter cell, but the link between the flagella (via the flagella connector) is released (Fig. 1G). We call this connection a ‘cytoplasmic bridge’ (see later). Abscission then follows to produce two uniflagellated daughters.

### Cell division in the bloodstream form

Bloodstream form cells also have a single attached flagellum that exits the flagellar pocket at the posterior end of the cell (Fig. 1I). The primary external morphological difference to the procyclic form is a flagellar pocket positioned closer to the posterior. Again, the first indication of cell division is the emergence of a short new flagellum that extends from a flagellar pocket (Fig. 1J; arrow) positioned posterior to the old flagellum (Fig. 1K). As in the procyclic form the flagella begin to segregate, and are more widely separated near the cell posterior (Fig. 1K). The new flagellum is attached to the cell body, but its distal tip is not connected to the old flagellum as in the procyclic form; instead it lies close alongside the old flagellum at it grows (Fig. 1L; dashed circle) with its distal tip embedded in an indentation of the cell body, called the groove (Hughes *et al*., 2013). Subsequently, the two flagellar pockets segregate and the flagellar pocket of the new flagellum is positioned posterior to and to the left of the flagellar pocket of the old flagellum when viewed from the cell's posterior. The new flagellum is positioned to the left of the old when viewed from the cell's posterior as in the procyclic form (Fig. 1K and L).

We can define the same four characteristic stages of cytokinesis in the bloodstream form as the procyclic: division fold generation, division furrow ingression, pre‐abscission and abscission. Fig. 1M illustrates a cell stage where the division fold is present (arrow), but the distal tip of the long new flagellum has grown unattached from the cell body. This is a cell stage that is not found the procyclic from where the distal tip of the new flagellum remains attached until the pre‐abscission stage. It was difficult to view more of the division fold in the bloodstream form due to the more ‘twisted’ nature of these cells. Two distinct posterior ends (Fig. 1M; circled) are evident, but the fold finishes closer to the pre‐existing posterior end in a bloodstream form cell than in a procyclic form cell (compare Fig. 1F with M). This difference makes it more difficult to reliably distinguish the old‐flagellum daughter cell from the new‐flagellum daughter cell at later time points (Fig. 1N and O). In Fig. 1N the division furrow has progressed towards the posterior end of the cell (arrow). Given that there is no flagella connector in bloodstream forms then this leads to a clearly bifurcated cell shape earlier in division than for the procyclic. Subsequently, a pre‐abscission stage exists where nascent daughters are apposed and joined at or close to their posterior ends (Fig. 1O). The cytoplasmic bridge connection is morphologically different to that typically observed procyclic form (compare Fig. 1H with O) in that it is broader and more substantial, although a similar substantial cytoplasmic bridge was observed on rare occasions in the procyclic. Abscission then follows (usually – see later) to produce two uniflagellated daughters.

### Division fold generation is unidirectional and requires precise insertion of microtubules between the two flagella

**Figure 2:**
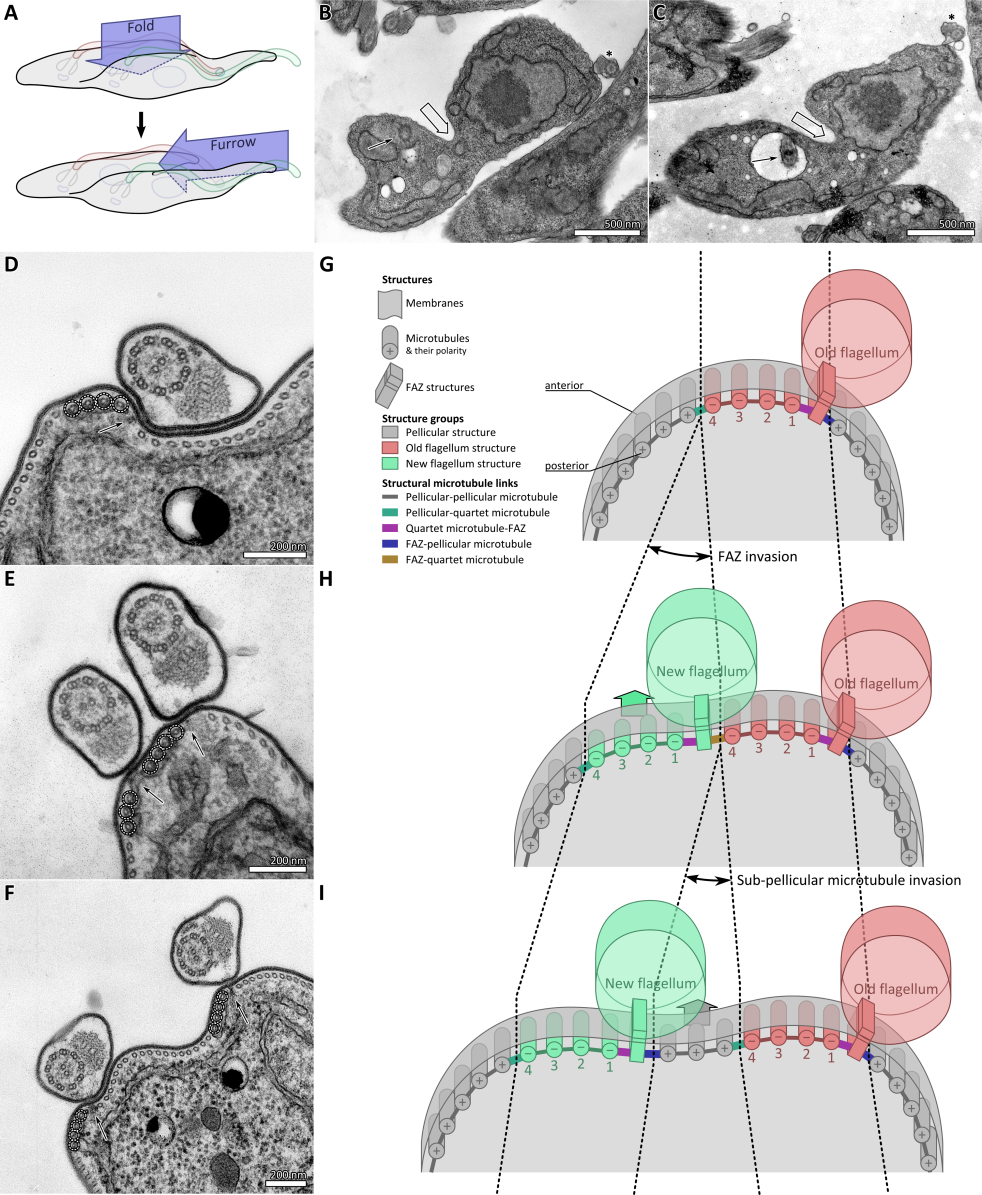
Microtubule‐mediated flagella segregation and unidirectional division fold ingression. A. Cartoon illustrating the ingression of the division fold and furrow. A procyclic form cell is shown, but the same structures arise in the bloodstream form during cytokinesis. B and C. Two examples of thin section transmission electron micrographs (TEMs) of transverse sections through PCF cells during unidirectional division fold (large open arrow) formation. The old flagellum is indicated with an asterisk and the basal body (in B), or flagellum within the flagellar pocket (in C), at the proximal end of the new flagellum are indicated with an arrow. D–F. Thin section TEMs of transverse sections through bloodstream cells at different stages of new flagellum and FAZ growth. FAZ filaments (arrows) and the microtubule quartet (MtQ, dashed circles) are indicated. (D) Section with a single flagellum closely associated with the FAZ. (E) Section with two flagella closely associated with their respective FAZ. There are no sub‐pellicular microtubules between the old FAZ MtQ and the new FAZ. (F) TEM of a transverse section through a bloodstream form cell with two segregated flagella closely associated with their respective FAZ. Indentation of the plasma membrane (large arrow) is unidirectional. G–I. Cartoon models of insertion of the new FAZ filament and MtQ among the sub‐pellicular microtubules, approximately corresponding to the micrographs in D–F. (G) Cartoon structure of a normal G1 phase single (old) flagellum and FAZ. Note the opposing polarities of the sub‐pellicular microtubules and the MtQ. Three distinct putative molecular‐scale structural linkages (pellicular microtubule‐MtQ, MtQ‐FAZ and FAZ‐pellicular microtubule) may be predicted. (H) Cartoon structure following invasion of the growing FAZ and microtubule quarter associated with the new flagellum. Invasion occurs along the pellicular microtubule ‐MtQ seam. This introduces fourth distinct putative molecular‐scale structural linkage; FAZ‐MtQ. (I) Cartoon structure following invasion of sub‐pellicular microtubules to segregate the new and old flagella. Invasion occurs along the FAZ‐MtQ seam. This restores FAZ‐pellicular microtubule and pellicular microtubule‐MtQ linkages. Note that either microtubule sliding or minus end sub‐pellicular microtubule polymerization is required for sub‐pellicular microtubule invasion.

We found that the first two stages of cytokinesis in procyclic and bloodstream forms were the generation of the lateral fold (Fig. 1D and M) followed by furrow ingression from the anterior to posterior along the line of the fold (Fig. 1F and N), both of which occur along a line between the old and new flagella (Fig. 2A). Although placement of the division fold was different between the life cycle forms, in that the fold finished closer to the pre‐existing posterior end in a procyclic form cell, its placement between the two flagella required segregation of the two flagella in both forms. As the sub‐pellicular array remains present at all division stages this would be expected to require insertion of microtubules between the old and new flagellum.

We carried out an in‐depth analysis of TEM cross‐sections of old and new flagella that were either still close to each other (i.e., where no segregation had occurred, as in Fig. 2E) or where segregation of the flagella was underway (Fig. 2F) in both life cycle forms. Prior to segregation the old and new flagella and flagella attachment complexes were closely associated in both forms (Fig. 2E). The four specialized microtubules [called the microtubule quartet (MtQ)] (Taylor and Godfrey, 1969; Lacomble *et al*., 2009) were associated with each flagellum and were located alongside one another (Fig. 2E; circled). When segregation was underway sub‐pellicular microtubules inserted precisely between the MtQ sets (Fig. 2F). A model outlines the insertion of sub‐pellicular microtubules between the two flagella for both forms (Fig. 2G–I). This demonstrates that initial invasion must be made between microtubule 4 of the MtQ of the old flagellum and the new flagellum attachment zone (FAZ) filament of the new flagellum (Fig. 2E), representing a unique seam. Further microtubule insertion between old MtQ microtubule 4 and the neighbouring sub‐pellicular microtubule highlights a second unique seam where polarity of MtQ number 4 is opposite to that of the neighbouring sub‐pellicular microtubule (Fig. 2F).

In cross‐sections of dividing cells, invagination of membrane to generate the division fold was always unidirectional in both forms – in‐folding occurred between the two flagella, but was never observed in the opposing area of the cell. No specific structural component was observed at the TEM level which could orchestrate indentation of the plasma membrane (Fig. 2B and C). No alteration of the normal spacing between sub‐pellicular microtubules was observed in the area of the fold or furrow.

### There are distinct areas of microtubule growth and re‐modelling during cell division

**Figure 3:**
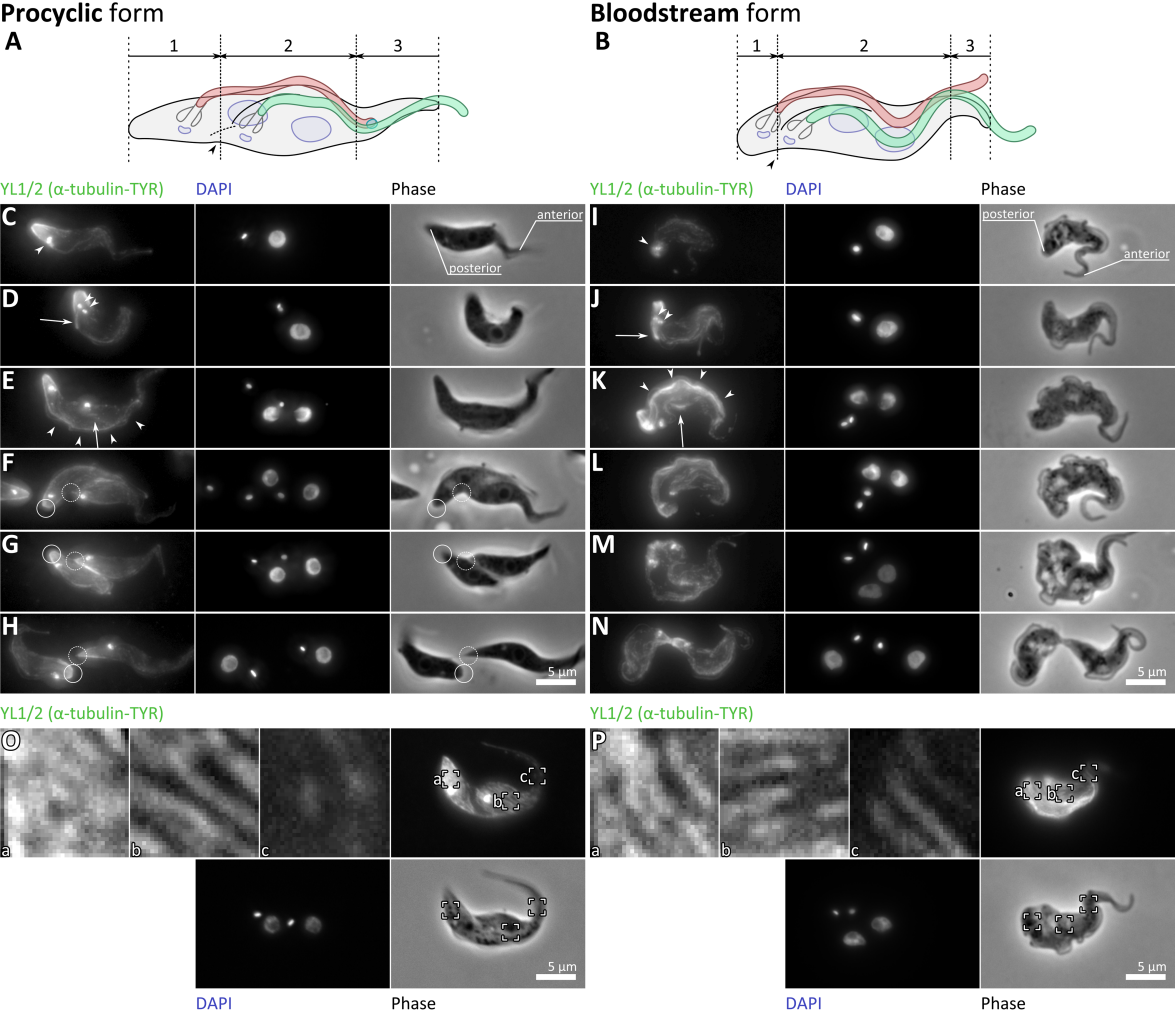
Differences in microtubule remodelling during cell division visualized by tubulin detyrosination dynamics. A and B. Cartoon representations of three zones of procyclic (A) and bloodstream form (B) cells that undergo distinct growth and morphogenetic events. Zone 1 (the posterior) is inherited by the new‐flagellum daughter cell. Zone 2 (the centre) is the dynamic area during division. Zone 3 (the anterior) is inherited by the old‐flagellum daughter cell. The forming new posterior end is indicated with an arrowhead. C–N. Maximum projections of YL1/2 (anti‐tyrosinated α‐tubulin) immunofluorescence, DAPI fluorescence and phase contrast micrographs of procyclic (C–H) and bloodstream (I–N) form cells during the cell division cycle. The basal bodies (arrowheads in C, D, I, J), new growing flagellum (arrows in D, J), mitotic spindle (arrows in E, K) and a region near the flagella during mitosis (arrowheads in E, K) are strongly labelled by YL1/2 in addition to regions of the sub‐pellicular microtubule array. The forming new (dashed circle) and old (solid circle) posterior ends of the procyclic form are indicated. O and P. Detail of YL1/2 labelling of posterior (a), central (b) and anterior (c) portions of the sub‐pellicular array in G2/cytokinetic procyclic (O) and bloodstream (P) form cells.

Our SEM analysis indicated cytokinesis involves an asymmetric fold whose placement differs between procyclic and bloodstream forms. The asymmetric fold placement means that different portions of the existing cell would be inherited by each daughter. We define three zones that undergo distinct cell morphogenetic events and inheritance in both life cycle forms: Zone 1 at the posterior of the mother cell would be inherited by the new‐flagellum daughter cell (Fig. 1F and M; Fig. 3A and B; labelled 1). Zone 2 in the middle (Fig. 1F and M; Fig. 3A and B; labelled 2) of the dividing trypanosome is an area of great complexity. It runs from a line close to the emergence of the new flagellum from the flagella pocket to the most anterior point of connection of the new flagellum to the cell body. In this middle zone three morphological features were observed to occur during cell division; (1) the division fold is generated; (2) a distinct morphing of the cell body to produce the new posterior end destined for the old‐flagellum daughter cell (Fig. 1F and M; Fig. 3A and B; arrowheads) a new anterior end must form which would be destined for the new‐flagellum daughter cell. The anterior zone (zone 3: Fig. 1F and M; Fig. 3A and B; labelled 3) would be inherited by the old‐flagellum daughter cell. These three zones are present in both bloodstream and procyclic forms but are of different sizes, with the positioning of the asymmetric division fold apparently linked with the position of the flagellar pockets and the distal tip of the new flagellum influencing the size of these zones (Fig. 1F and M; Fig. 3A and B).

The sub‐pellicular microtubule cytoskeleton remains intact during division and defines cell shape, therefore we analysed how the microtubules are rearranged during cell division. Although we know that this re‐modelling involves insertion of new microtubules between old ones and elongation of existing microtubules (Sherwin and Gull, 1989b; Robinson *et al*., 1995) precisely when and where this occurs in the cell cycle is not known. We have characterized temporal microtubule dynamics in the procyclic form by labelling cells with the YL1/2 antibody, which specifically recognizes the C‐terminal tyrosine of α‐tubulin (Sherwin and Gull, 1989a). This tyrosine is removed by a tyrosine carboxypeptidase following assembly of the tubulin heterodimer into a microtubule therefore tyrosinated α‐tubulin is a marker of newly formed microtubules (Kilmartin *et al*., 1982; Sherwin and Gull, 1989a). This analysis was repeated in more detail and extended to the bloodstream form to analyse how microtubule dynamics link to morphogenesis in the cell cycle. The sub‐pellicular microtubules have the same polarity, with their plus ends at the posterior end of cell and minus ends at anterior end of the cell (Robinson *et al*., 1995). The posterior end of the cell was labelled throughout all of the cell cycle (Fig. 3C and I; arrow), reflecting turnover of tubulin at the plus ends. The basal bodies at the base of the flagellum were also labelled in all cells, reflecting unmodified tubulin awaiting transport into the flagellum (Stephan *et al*., 2007) (Fig. 3C, D and I–J; arrowhead). As the cell division cycle progressed and new flagellum growth began it was also labelled, due to the addition of tubulin dimers at the plus end of extending microtubular axonemes (Fig. 3D and J; arrow). The mitotic spindle was also labelled (Fig. 3E and K; arrow).

During and following mitosis the complexity of labelled YL1/2 structures increased, reflecting a change in the pattern of microtubule dynamics in preparation for cytokinesis. Three distinct patterns appeared. During mitosis one side of the cell, near the old and new flagella, was strongly labelled (Fig. 3E and K; arrowheads). This may be associated with invasion of new microtubules during flagella segregation. Following mitosis the central portion of the cell was extensively labelled in a striated pattern in both life cycle forms (Fig. 3F, G, L and M). In the procyclic form, where zones 1 and 3 are large (Fig. 1F and Fig. 3A in comparison to Fig. 1M and Fig. 3B), the pattern of labelling in zones 1, 2 and 3 were distinct; with strong, even, labelling, striated labelling, and weak labelling respectively (Fig. 3O). Logically this suggested an increase in sub‐pellicular microtubule array length in zone 1 and an increase in width in zone 2, but no growth in zone 3, during cytokinesis. In the bloodstream form the striated labelling extended closer to the anterior and posterior (Fig. 3P), consistent with striated labelling being associated with zone 2. During late cytokinesis, formation of a nascent posterior end for the old‐flagellum daughter was clearly observed in the procyclic form (Fig. 3G and H; dashed circles). Finally, pre‐abscission stages in both life cycle forms (Fig. 3H and N) had a similar pattern of YL1/2 staining to the respective G1 cell (Fig. 3C and I). These results clearly demonstrate the asymmetric re‐modelling events of the sub‐pellicular microtubule cytoskeleton required to achieve an asymmetric division fold and inheritance of microtubules to different daughters.

### XMAP215 distribution reveals the dynamics of microtubule remodelling at the cell posterior

**Figure 4:**
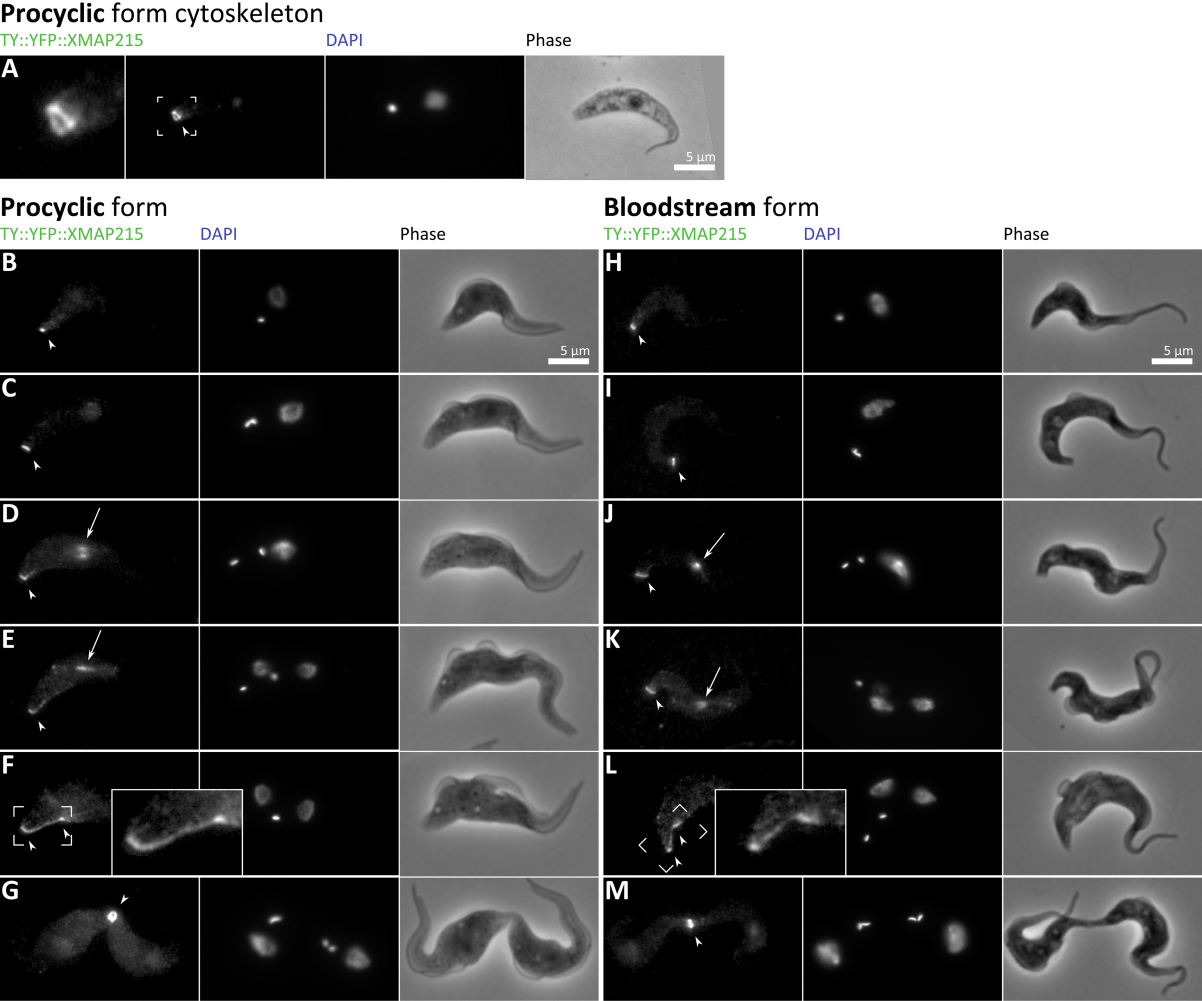
Morphogenesis of the cell posterior visualized by the tip‐tracking microtubule polymerase XMAP215. A. Detail of YFP::XMAP215 localization at the posterior of a detergent‐extracted G1 procyclic form cell. B–M. Native fluorescence of YFP::XMAP215, DAPI fluorescence and phase contrast micrographs of procyclic (B–G) and bloodstream (H–M) form cells during the cell division cycle. The cell posterior (arrowheads) and mitotic spindle (arrows) are indicated and an inset shows the structure of the forming new posterior end during cytokinesis (F, L).

We have established that there is formation of a new posterior end for the old‐flagellum daughter in both bloodstream form and procyclic forms. It is clearly a key morphogenetic stage in the cell division cycle of both life cycle forms and must coincide with formation of the division fold during cytokinesis. This process must incorporate microtubule re‐modelling to bring together a bundle of aligned plus ends of microtubules at the posterior end of the cell (Robinson *et al*., 1995). To understand this further we created an N‐terminal YFP fusion of the endogenous *T. brucei* homologue of the well characterized microtubule plus end binding protein XMAP215 (Asbury, 2008). This was done in both life cycle forms to detect microtubule re‐modelling during posterior end formation (Fig. 4). Early in the cell division cycle a discrete patch of labelling was observed at the very posterior end of the cell in both life cycle forms (Fig. 4A, B and H; arrowhead) reflecting where microtubule plus ends are concentrated. In detergent‐extracted cytoskeletons labelling at the posterior end appeared ring‐shaped (Fig. 4A). As the cell division cycle progresses the discrete patch was more elongated and may represent an alteration in microtubule bundling at the posterior end to allow for re‐modelling (Fig. 4B, C, J and K). During mitosis additional labelling was also observed in the nucleus and spindle (Fig. 4D, E, J and K; arrows) as expected for XMAP215.

Post‐mitosis two discrete patches of labelling were observed at each of the nascent posterior ends. One at the posterior end of the cell and one further from the posterior end, potentially labelling the position of the nascent posterior end of the old‐flagellum daughter, with a line of labelling connecting them (Fig. 4F and L). In the bloodstream form we generally observed this pattern of labelling only when XMAP215 was no longer detectable in the nucleus (Fig. 4 L), but in the procyclic form this new pattern of labelling was already apparent when XMAP215 was still detectable in the nucleus (Fig. 4E), and remained after mitosis (Fig. 4F). At the pre‐abscission stage the posterior ends of both daughter cells were labelled. Notably no other regions of concentrated microtubule plus ends are seen elsewhere in the sub‐pellicular microtubules at any cell cycle stage in either life cycle form, including in regions associated with flagella segregation or fold generation or furrow ingression. We noted a propensity for cells to delay or fail abscission and re‐enter the cell division cycle, which may be due to expression of YFP‐XMAP215. In these cases, bright patch of labelling was found at the posterior end of each daughter (Fig. 4G and M). In conclusion, XMAP215 has demonstrated the formation of bundles of microtubule plus ends at the two posterior ends during cell division. The positions of these bundles indicate they arise through different pathways; (1) re‐modelling of the existing posterior end and (2) formation of a new posterior end in the side of the sub‐pellicular array. The former is positioned such that the cytoskeletal components may directly inherit morphogenetic cues from the organization of the pre‐existing posterior end, while the latter could not. The inheritance of microtubules of the sub‐pellicular array is semi‐conservative (Sherwin and Gull, 1989b) and, as the generation of the nascent posterior ends involves cytoskeletal reorganization, it seems likely the inheritance of cytoskeletal components in the nascent posterior ends is semi‐conservative too.

### Daughter cells are asymmetric in posterior end morphology at abscission and undergo post‐abscission morphogenesis

**Figure 5:**
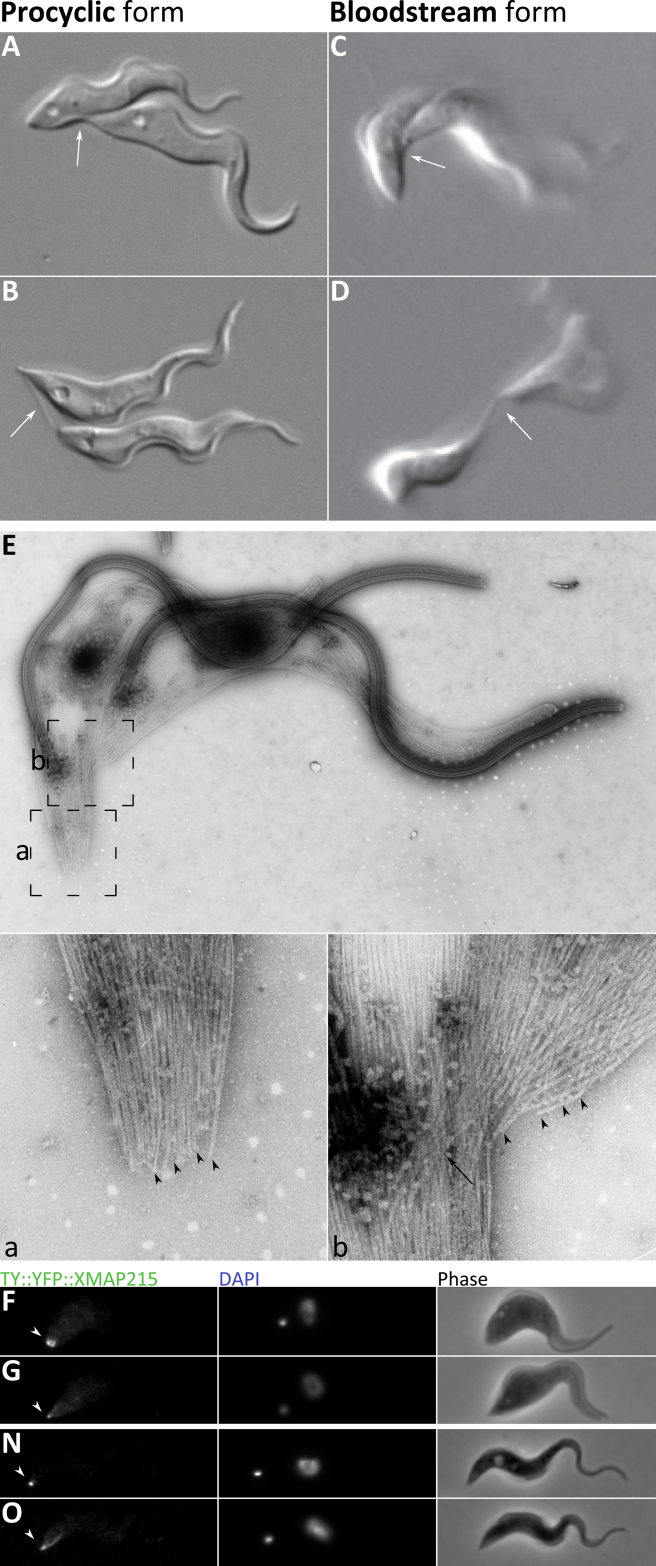
Cytoplasmic bridge and abscission. A–D. Still images from DIC videomicroscopy of live dividing procyclic (A, B) and bloodstream (C, D) form cells, connected by a cytoplasmic bridge (arrow). E. TEM of a whole mount cytoskeleton of a pre‐abscission procyclic form cell. Detail of the old and newly forming posterior are shown with arrowheads indicating examples of microtubule plus ends, and an arrow indicating a bundle of microtubules which extend from the newly forming posterior end into the portion of the sub‐pellicular array which will be inherited by the daughter cell with the new flagellum. F–O. Examples of XMAP215 labelling of G1 procyclic (F, G) and bloodstream (N, O) cells with a more rounded (F, N) or pointed (G, O) posterior end.

Our SEM analysis demonstrated in both life cycle forms that during the pre‐abscission stage the two nascent daughter cells remain connected via a cytoplasmic bridge – a thin section of membrane linking the posterior end of the old‐flagellum daughter cell (Fig. 1H and O; circled) with the side of the new‐flagellum daughter cell. This can be seen clearly by live cell imaging in both bloodstream and procyclic forms, demonstrating the strength of the connection between the daughter cells at this stage (Movies S1 and S2, Fig. 5A–D, arrows). We frequently observed twisting of the two daughter cells when furrow ingression had reached the posterior end of the fold and both daughter cells are attached at or close to their posterior ends prior to abscission (Fig. 1O and Movie S3). Analysis of detergent‐extracted cytoskeletons revealed that in the pre‐abscission stage, while many microtubules terminate to form the new posterior end (Fig. 5E arrowheads and inset a), a small number continue in length and were found to still be integrated in the sub‐pellicular array up to the old posterior end (Fig. 5E and inset b). This is consistent with XMAP215 labelling (Fig. 4F and L) showing a line of microtubule plus ends between the two nascent posterior ends and together suggests that the cytoplasmic bridge contains microtubules.

The distinct organization of the pre‐abscission cell means that the nascent posterior end of the new‐flagellum daughter, particularly in the procyclic form, has a ‘rounded end’ profile (Fig. 1G–H and Fig. 5A) while the nascent posterior end of the old‐flagellum daughter has a ‘pointed end’ profile (Fig. 1G–H and Fig. 5A). In supplementary data Movie S1 abscission occurs and the two different posterior end profiles (one rounded and one pointed) were clearly evident pre‐abscission and following abscission as the two daughters moved away from each other; thus the two daughters are not identical at abscission. XMAP215 labelling also indicated two distinct organizations of microtubule plus ends found at the posterior of both procyclic (Fig. 5F and G) and bloodstream (Fig. 5N and O) forms; most commonly in a small bundle which may be resolved as a ring in some cells (Fig. 5F and N) but occasionally as a tighter focus of labelling associated with a more pointed posterior. The latter is likely to reflect a cell shortly post‐abscission which inherited the newly formed posterior.

This difference in posterior end structure post‐abscission implies the existence of post‐division morphological changes to produce a rounded posterior end in the old‐flagellum daughter cell. At pre‐abscission the cytoplasmic bridge was sometimes found at a more posterior position, leading to a more ‘end to end’ morphological configuration of the two daughter cells (compare the position of the cytoplasmic bridge connection in Fig. 1G versus H, and between Fig. 5A and B). This could represent a later time point in pre‐abscission as the thin section of membrane relocated towards the posterior end of the other daughter cell before release of two daughter cells with the onset of cell abscission. The two daughters remained attached via the cytoplasmic bridge connection for a significant length of time in both life cycle stages (see, for example, supplementary Movie S3, where the two daughters remained attached for 24.5 min before abscission), illustrating the significance of this stage in cell division cycle timings.

### Bloodstream form trypomastigotes often re‐enter the cell cycle before final abscission

**Figure 6:**
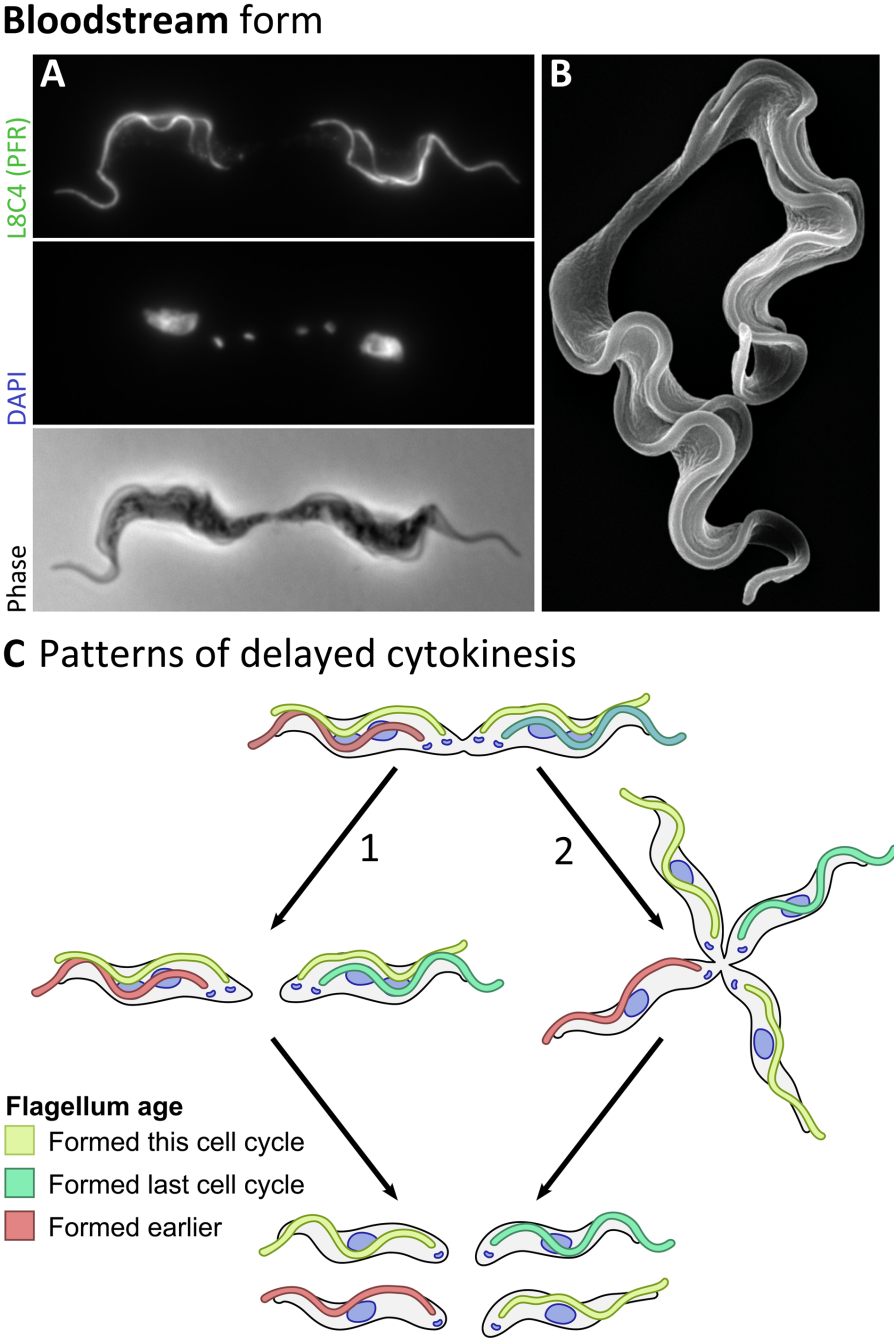
Quadriflagellate doublet cells. A. SEM of quadriflagellate doublet cells in the bloodstream form. Daughter cells are attached by their posterior ends and both possess two flagella. B. L8C4 (anti‐PFR) immunofluorescence, DAPI fluorescence and phase contrast micrographs of a quadriflagellate bloodstream form cell with 4 kinetoplasts, 4 nuclei and 4 flagella. C. Cartoon depicting two hypothetical routes to cell division of quadriflagellate doublet cells resulting in 4 1K1N cells. The 2K2N cells generated by route 1 would be morphologically indistinguishable from 2K2N cells from the normal cell cycle.

In our SEM analysis we identified an additional morphological cell type in the bloodstream form, where daughter cells were still attached by their posterior ends, but each possessing a second flagellum; indicating they had either prematurely re‐entered the cell cycle before abscission or failed abscission. We called these ‘quadriflagellate doublet cells’ (Fig. 6A and B). Individuals comprising these quadriflagellate doublet cells were synchronous (as defined by the number of kinetoplasts, nuclei and flagella) in their cell cycle positioning. Our counts revealed that this cell type represented a significant proportion (1.5%) of the population (*n* = 1000 cells) in our in vitro cultured cell line. The cartoon in Fig. 6C depicts two hypothetical pathways for cytokinesis of the quadriflagellate doublet cells. Importantly, in pathway 1 two 2K2N cells could be produced (Fig. 6C), which would be morphologically indistinguishable from 2K2N cells in the first cell cycle and may mask the true extent of the contribution of this pathway to proliferation in the bloodstream form. Similar quadriflagellate doublet cells were observed occasionally in the procyclic form, but were much less common; therefore these quadriflagellate doublet cells could have implications for the differing impact of mutations on division phenotypes in the two life cycle stages.

## Discussion

In this paper we have carried out an in‐depth analysis of cytokinesis in *Trypanosoma brucei* and a comparison of cell morphogenesis in the two major life cycle forms (bloodstream and procyclic) that are widely used in molecular and RNAi studies. We provide the most detailed analysis of microtubule‐driven cytokinesis in this important protist, and the morphogenetic differences between the two life cycle stages.

We have characterized four morphological stages of cytokinesis in trypanosomes; division fold generation, division furrow ingression, pre‐abscission and abscission. Positioning of the division furrow is a key factor in eukaryotic cell cytokinesis and there is not a common mechanism amongst eukaryotic organisms for the selection of the division site. In animal cells the mitotic spindle is important for division site placement (Glotzer, 2009), in yeast the previous site of budding is the marker for the next division site (Pollard and Wu, 2010) and in plants a microtubule‐based pre‐prophase band marks the site for division (Van Damme, 2009). In *T. brucei* we show that the position of the division fold and path of ingression of the furrow is positioned asymmetrically along the long axis of cell, and is more asymmetric in the procyclic form. The anterior end of the division fold coincides with the anterior end of new flagellum attachment to the cell body, and we have previously hypothesized that the flagellum attachment zone (FAZ) is involved in defining the point at which furrow ingression starts (Robinson *et al*., 1995; Vaughan *et al*., 2008; Farr and Gull, 2012). We found no other morphological clues in the structure of the sub‐pellicular array for asymmetric placement of the division fold along the long axis of the cell or the position of the newly forming posterior end. In *T. brucei* a single Aurora kinase localizes along between to the two nuclei and kinetoplasts post‐mitosis, which is very likely locating the position of the division fold for cytokinesis and is part of a distinct chromosomal passenger complex (CPC) composed of two novel kinesins (Li *et al*., 2008a,b).

As described in the introduction trypanosomes do not have filamentous actin or the actin/myosin molecular machinery. Cleavage of two daughters occurs via the generation of a fold followed by unidirectional furrow ingression along the fold. Fold formation by in‐folding of the plasma membrane between the two flagella increases the cell surface therefore an increase in quantity of plasma membrane, sub‐pellicular microtubules and microtubule‐membrane and microtubule‐microtubule attachments is expected. Formation of the division fold was not associated with a particular pattern of YL1/2 or XMAP215 labelling suggesting little deviation from the normal sub‐pellicular microtubule array organization in this region and, as the in‐folding is a simple deformation of cell shape and runs along a line parallel to the sub‐pellicular microtubules, no growth or disassembly of the microtubules is logically required to achieve fold generation. Our TEM cross‐sections demonstrated addition of sub‐pellicular microtubules and in‐folding of the plasma membrane between the old and new two flagella, specifically a site between the old and new FAZ. Again, this highlights the important role played by the FAZ as a positional marker for cytokinesis. There was no change in microtubule spacing or any specific ultrastructural component at the site of in‐folding. The unidirectional nature of fold generation between the two flagella is intriguing and would be expected to require some means of force generation. This could be generated in one of many ways, new microtubules insertion, alteration of the angle between sub‐pellicular microtubule cross‐linkages, high intrinsic curvature associated with the two FAZ structures or increase in surface area in the absence of increased cell volume.

Following generation of the division fold, furrow ingression begins at the anterior end of the fold, and progresses towards the posterior in the bloodstream form. In the procyclic form the flagella connector prevents unimpeded observation of this process, although it ingresses from the anterior of the fold so seems likely to occur through a similar mechanism. Furrow ingression does not change the topology of the plasma membrane; therefore no membrane fusion events are logically necessary for ingression. Like the fold, formation of the furrow was not associated a particular pattern of YL1/2 or XMAP215 labelling and, as the furrow appears to ingress along the long axis of microtubules, no growth or disassembly of the microtubules is logically required to achieve furrow ingression, although remodelling of microtubule‐microtubule cross‐linkages is necessary to achieve furrow ingression.

In order to resolve cytokinesis there must be a point where microtubules are either cut or extracted from the sub‐pellicular array and a membrane fusion event must occur in order to separate the two connecting daughters at the pre‐abscission stage. In animal cells furrow ingression occurs at the spindle midzone resulting in a narrow opening between the daughters called the cytoplasmic bridge. This is composed of overlapping anti‐parallel microtubules of the mitotic spindle and an electron dense midbody structure. During this pre‐abscission stage membrane components are delivered via ESCRT proteins to allow for final scission and the process is regulated by Aurora kinase B (Steigemann and Gerlich, 2009). This pre‐abscission stage, when the two daughters remain connected via the cytoplasmic bridge can vary in times between minutes to many hours in Ptk2 cells (Sanger *et al*., 1985). Further work is required to confirm the role of microtubules and presence of other components for abscission at the cytoplasmic bridge in trypanosomes (Farr and Gull, 2012), but presence of Aurora kinase at the site where the cytoplasmic bridge is formed suggests a conserved mechanism of abscission regulation (Steigemann and Gerlich, 2009).

The cytoplasmic bridge structure in the bloodstream was found to be morphological different to that in the procyclic form. This may arise from to the smaller asymmetry in the formation pathway and shape of the bloodstream form posterior ends and may lead to our observation that bloodstream form cells often remaining connected via the cytoplasmic bridge connection while re‐initiating S‐phase, which we have called quadriflagellate doublet cells. Cell types reminiscent of the quadriflagellate doublet cells have been described in classical descriptions of trypanosomes in the original literature of African trypanosomes *Trypanosoma simiae*, *T. brucei* and *T. congolense* (Bruce *et al*., 1912; Wenyon, 1926; Soltys and Woo, 1969; Ormerod and Venkatesan, 1971) and the rodent trypanosome *T. lewisi* in samples derived from animal infections (Wenyon, 1926; Wolcott, 1952; Ormerod and Killick‐Kendrick, 1956; Hoare, 1972). Delayed abscission has also been described in cultured promastigote *Leishmania mexicana*, a related trypanosomatid parasite, where nearly 10% of the population appears to have delayed abscission, but otherwise progresses through the cell cycle normally (Wheeler *et al*., 2011). Failure to complete abscission is not peculiar to trypanosomatids and has been found in specialized cell types in metazoan such as ring canals that connect Drosophila egg chambers and in spermatogenesis where intercellular bridges remain connected (Magie *et al*., 1999; Greenbaum *et al*., 2006: 200). The potential function of delayed abscission in the bloodstream is unclear, especially as comparatively little is known about the precise behaviours of parasites in the blood. These doublet cells are clearly motile, however it seems likely their ability to undergo directed motion would be impaired; this may have interesting consequences for the ability to perform hydrodynamic‐assisted recycling of the surface coat (Engstler *et al*., 2007).

**Figure 7:**
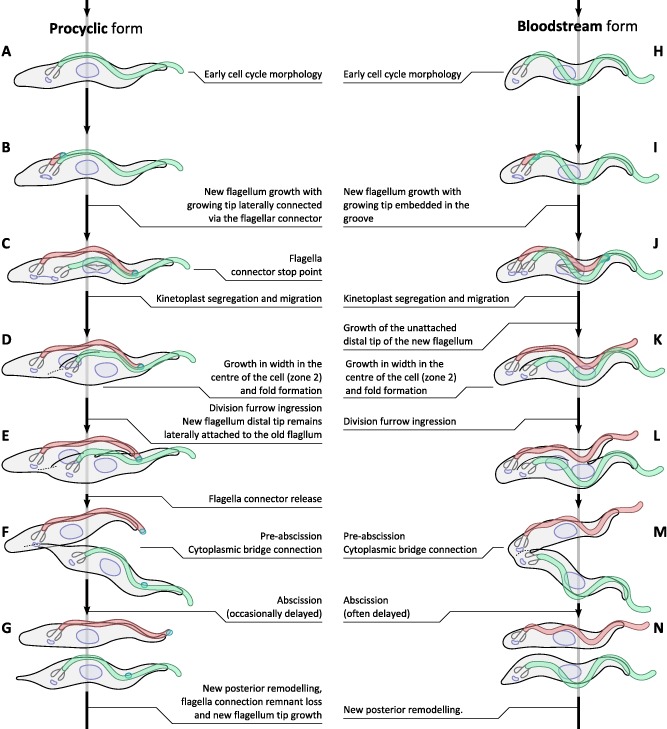
Differences in morphogenesis of the bloodstream and procyclic forms of *T. brucei* through the cell division cycle. Major morphogenetic events are shown alongside cartoon representations of the associated cell morphologies. Vertical spacing is purely illustrative and does not represent relative time spent during each of these stages.

At abscission our study also showed that the posterior ends of the two daughters, particularly in procyclic forms, are morphologically different (pointed versus rounded) arising from underlying microtubule organization resulting from resolution of the cytoplasmic bridge. Indeed in mammalian cells asymmetry in newly divided cells is also receiving attention and it is now known that the midbody structure of the cytoplasmic bridge is inherited by only one of the two daughter cells at abscission (for review see (Barr and Gruneberg, 2007). This asymmetry implies the pointed end profile must be re‐modelled after abscission, which is the second post‐division morphogenetic event identified in procyclic forms in addition to a small extension of the new flagellum beyond the end of the cell body after abscission (Fig. 7G) (Farr and Gull, 2009).

The procyclic and bloodstream form cell cycles, while overall similar, have several differences significant for understanding the biology of the parasites, summarized Fig. 7. The overall morphology of the bloodstream and procyclic life cycle stages is similar; both are trypomastigotes with the kinetoplast positioned posterior to the nucleus and a laterally attached flagellum, the defining features of the trypomastigotes (Fig. 7A and H) (Hoare and Wallace, 1966). The procyclic form is longer, wider, with a shorter portion of the flagellum overhanging the cell anterior, a less contorted cell shape and a kinetoplast positioned further from the posterior than in the bloodstream form. These are all modulations of the same underlying cell organization. In the bloodstream form the new flagellum extends, with the distal tip located in the groove (Hughes *et al*., 2013), until the tip reaches (or is very close to) the end of the existing cell body (Fig. 7I and J). The new flagellum then continues to grow unattached to the cell body during cytokinesis (Fig. 7K and L). However, in the procyclic form the new flagellum grows with its distal tip attached to the old flagellum via the flagella connector (Moreira‐Leite *et al*., 2001) until the tip reaches a point ∼ 60% along the old flagellum, the ‘stop point’ (Davidge *et al*., 2006). New flagellum growth continues, maintaining complete flagellum attachment to the cell body, but the new flagellum tip moves no further relative to the old flagellum. This continued growth coincides with movement of the base of the flagellum, the flagellar pocket, and kinetoplast segregation (Fig. 7B and C) (Davidge *et al*., 2006; Absalon *et al*., 2007), which is more extensive in the procyclic form with a post‐mitotic KNKN (Fig. 7D) configuration of the kinetoplasts (K) and nuclei (N) along the posterior to anterior long axis of the cell than in the bloodstream form where the kinetoplasts remain close (KKNN) (Tyler *et al*., 2001) (Fig. 7K). These differences are likely linked to the function of the flagella connector, which has previously been hypothesized to be responsible for the stop point of the procyclic flagellar growth leading to kinetoplast segregation (Davidge *et al*., 2006).

There is arguably a single major structural difference relevant for division and morphogenesis between the procyclic and bloodstream forms: the presence of a flagella connector at the growing flagellum tip, in the procyclic form, or embedding of the growing new flagellum tip in the groove in the bloodstream form. The presence of the flagella connector, which only releases following division furrow ingression in the procyclic form, gives rise to a distinctive appearance difference between cells during furrow ingression and connector detachment an additional morphogenetic event in the procyclic form (Fig. 7E and F). In the bloodstream form there is a distinctive morphogenetic event where the groove must undergo a level of regulation to allow release of the distal tip and growth of the distal end unattached from the cell body prior to cytokinesis (Fig. 7K).

During division ours and previous analyses have shown the kinetoplasts segregate less far, the flagellum tip extends further, and the division furrow positioning is more symmetric in the bloodstream relative to the procyclic form. Again these reflect modulations rather than distinct differences. The presence or absence of a flagella connector stop point and the degree of segregation of the kinetoplasts appears linked to the positioning of the division fold and furrow. The position of the division fold and furrow therefore differs between the procyclic and bloodstream form cells with the bloodstream form undergoing more bilaterally symmetric cytokinesis, and the degree of asymmetry may be derived from the way in which the new flagella grow and template morphology of the daughter cells (Fig. 7F and M).

The major morphogenetic differences between the two trypomastigote forms are important to understand when comparing the results of functional studies in both life cycle forms, with particularly important implications for analysis of cell cycle defects in RNAi experiments. Differences in phenotype when expression of the same gene is ablated by RNAi in the procyclic and bloodstream forms are widely reported. For example, ablation by RNAi of RACK1 (receptor for activated kinase‐1) results in a partial division furrow ingression (referred to as partial cleavage) in the procyclic form, but no division furrow (referred to as cleavage furrow) detected in the bloodstream form (Rothberg *et al*., 2006). When motility is compromised the procyclic form is often still able to divide and are viable, but bloodstream form cells tend not to be able to divide when the same gene was ablated by RNAi (Branche *et al*., 2006; Broadhead *et al*., 2006; Ralston *et al*., 2006).

It is clear that distinct cell morphogenetic processes will need to be taken into account before conclusions can be drawn and, although both of these cell types are generally referred to as trypomastigotes (Hoare and Wallace, 1966), it has become increasingly clear that important differences exist and that this level of morphological classification may now be unhelpful.

## Experimental procedures

### Cell culture

Procyclic form *T. brucei brucei* Lister 427 cells were cultured at 28°C in SDM‐79 medium supplemented with 10% (v/v) heat‐inactivated fetal calf serum as described previously (Brun and Schönenberger, 1979). Bloodstream form *T. brucei brucei* 427 cells were cultured at 37°C in HMI‐9 medium supplemented with 15% (v/v) heat‐inactivated fetal calf serum.

### Transmission electron microscopy

Thin section and whole mount cytoskeleton electron microscopy samples were prepared as previously described (Lacomble *et al*., 2009). Micrographs were captured on a Hitachi H‐7650.

### Scanning electron microscopy

Scanning electron microscopy samples were prepared as previously described (Sharma *et al*., 2008). Images were captured on a JEOL JSM‐6390.

### Immunofluorescence microscopy

Cells were fixed in media to a final concentration of 2% formaldehyde in PBS before settling onto glass slides and permeabilized by immersion in −20°C methanol for 5 min then stained with the appropriate primary and secondary antibodies and 4,6‐diamidino‐2‐phenylindole (DAPI). Tyrosinated α‐tubulin was labelled with YL1/2 (Kilmartin *et al*., 1982), a monoclonal rat antibody kindly provided by J. Salisbury. The PFR was labelled with L8C4 (Kohl *et al*., 1999), a monoclonal mouse antibody.

### Bloodstream form population count

The approximate position of individual cells within the cell division cycle is readily determined by DAPI staining of the mitochondrial and nucleus DNA. L8C4 immunofluorescence samples were prepared from logarithmic bloodstream form culture. A count was performed on the proportion of cells attached at their posterior ends (assessed by phase contrast) with two kinetoplasts (assessed by DAPI), two flagella (assessed by L8C4) and no more than 4K4N and 4 flagella. This cell type was recorded as quadriflagellate doublet cells.

### Live cell imaging

Live cell micrographs and time lapse videomicrographs were captured using a Zeiss Axioplan microscope and a Photometrix Coolsnap HQ CCD camera using Metamorph software. A Bioptechs heated stage was used to maintain procyclic and bloodstream form cells at 28°C and 37°C respectively.

### Endogenous locus tagging

A cell line expressing Ty::YFP::XMAP215 fusion protein from the endogenous locus was generated using pEnT6P (Kelly *et al*., 2007) in *T. brucei* 427 SiMP (Daniels *et al*., 2012), which relies on endogenous readthrough transcription, as previously described.

## Supplementary Material

Supporting Information

Supporting Information

Supporting Information
